# Natural Bitter Compounds, Intestinal TAS2Rs, and GLP-1 Secretion: Mechanistic Evidence and Translational Challenges

**DOI:** 10.3390/ijms27219816

**Published:** 2026-11-01

**Authors:** Wenmin Lao, Xiaojing Liu, Jiefang Zhou, Fengmei Zhu, Jun Li, Wentao Xu, Yongsheng Liu

**Affiliations:** 1 College of Food Science and Technology, Hebei Normal University of Science and Technology, Qinhuangdao 066000, China; laowenmin93@163.com (W.L.); liuxiaojing6666@163.com (X.L.); fanerous@126.com (J.Z.); zhufengmei0714@163.com (F.Z.); 2 Department of Nutrition and Health, China Agricultural University, Beijing 100091, China; xuwentao@cau.edu.cn; 3 College of Animal Science and Technology, Hebei Normal University of Science and Technology, Qinhuangdao 066000, China; liuys4031@hevttc.edu.cn

**Keywords:** bitter taste receptor, natural bitter compounds, GLP-1, enteroendocrine cells, intestinal chemosensing, gut microbiota

## Abstract

Bitter taste receptors (TAS2Rs) are expressed beyond the oral cavity, and those in the intestine have been implicated in metabolic regulation. Selected natural bitter compounds (NBCs) increase glucagon-like peptide-1 (GLP-1) release in experimental enteroendocrine models, but predicted ligand binding, functional receptor activation, and receptor-dependent hormone secretion do not provide equivalent evidence. Intestinal TAS2Rs may thus provide a route to endogenous GLP-1 modulation distinct from receptor agonist therapy. Unlike broader reviews of extraoral chemosensing, this review maps these distinct endpoints for individual NBC-TAS2R pairs and separates measured GLP-1 release from changes in proglucagon expression. We define a receptor-supported enteroendocrine core and distinguish it from indirect evidence and testable hypotheses concerning microbiota-associated, immune–endocrine, and gut–brain interfaces. Receptor-directed perturbation supports selected compound-specific secretory responses, whereas the three proposed interfaces lack complete NBC-TAS2R-GLP-1 causal chains. We critically compare human-derived cell lines, animal models, primary tissues, and organoids, and summarize the experimental concentrations and GLP-1 assay endpoints where reported. Finally, we identify priorities for receptor-selective validation, measurement of intestinal target-site exposure, and prospective human studies. This evidence map clarifies what is experimentally supported and what must be demonstrated before intestinal TAS2Rs can be considered reliable targets for endogenous GLP-1 modulation.

## 1. Introduction

Metabolic diseases, particularly type 2 diabetes mellitus (T2DM) and obesity, represent a major global health burden [1]. The prevalence of diabetes is projected to continue rising over the coming decades [2], while obesity remains a major contributor to metabolic morbidity [3]. Current glucose-lowering therapies provide important clinical benefits but have treatment-specific limitations [4,5,6]. These limitations, together with the therapeutic success of incretin-based pharmacology, have renewed interest in endogenous intestinal mechanisms that regulate glucagon-like peptide-1 (GLP-1) secretion and may be modifiable by dietary or luminal signals.

**Figure 1:**
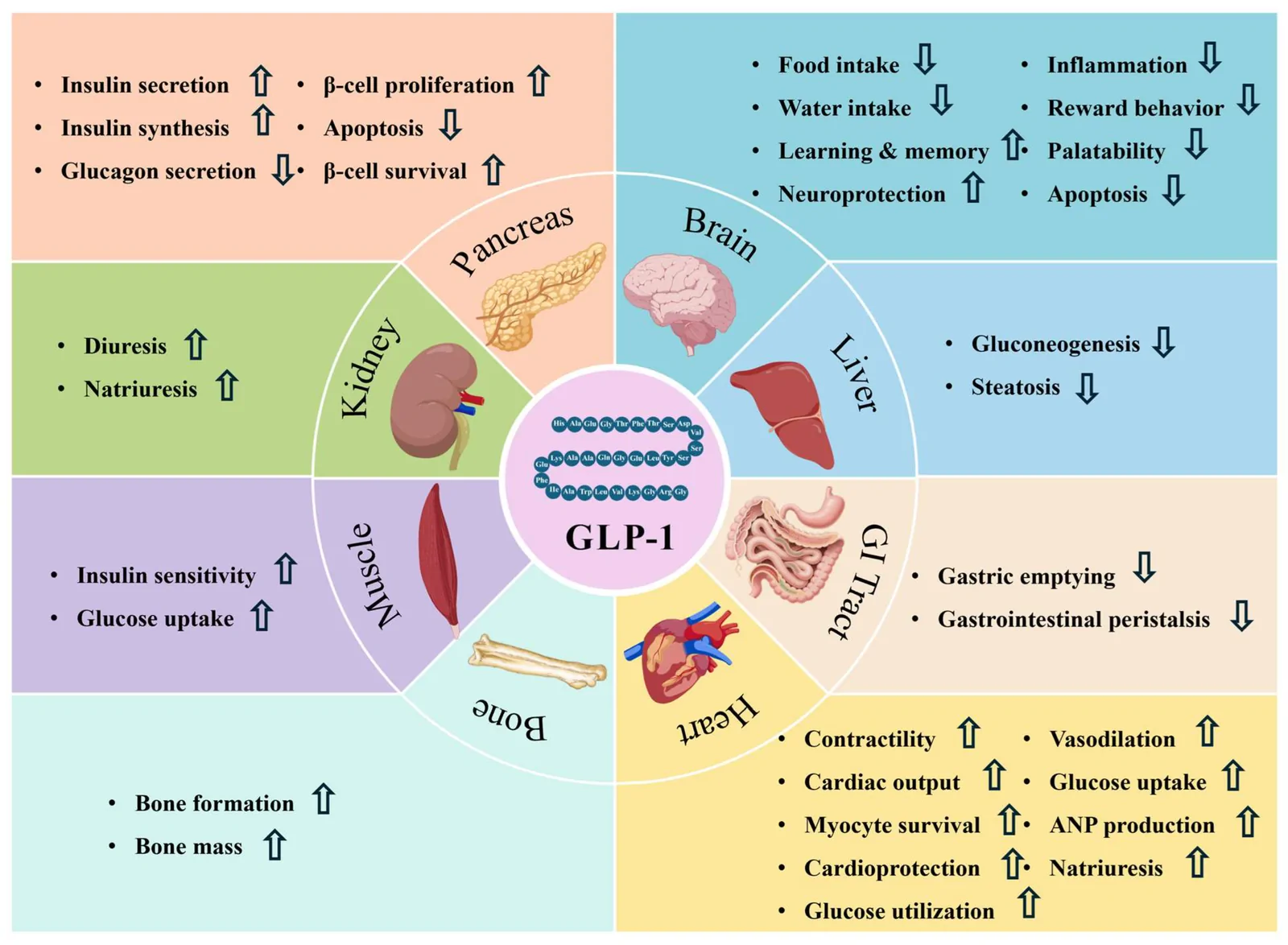
Selected reported effects associated with GLP-1 or GLP-1 receptor signaling. Upward arrows (↑) indicate an increase or enhancement, whereas downward arrows (↓) indicate a decrease or reduction. GI denotes gastrointestinal, and ANP denotes atrial natriuretic peptide. Created with BioGDP.com [24].

GLP-1, a hormone secreted by intestinal enteroendocrine L-cells, contributes to glucose and energy homeostasis [7]. As an incretin hormone, GLP-1 enhances glucose-dependent insulin secretion and participates in appetite and body-weight regulation [8,9,10,11]. In addition, GLP-1 receptor signaling in pancreatic and selected extra-pancreatic tissues contributes to glycemic regulation and broader physiological effects [12]. Selected effects associated with GLP-1 and its receptor signaling are summarized in Figure 1. Native GLP-1 is rapidly inactivated in the circulation [13,14], whereas pharmacological GLP-1-based therapies prolong incretin activity [15,16]. GLP-1 medicines are highly effective but can produce gastrointestinal adverse effects [16,17].

Bitter taste receptors (TAS2Rs) are G protein-coupled receptors (GPCRs) originally identified in oral bitter taste perception [18,19], but several subtypes are also expressed in the gastrointestinal tract [20,21,22,23]. Their intestinal expression makes them candidates for sensing luminal chemicals, although receptor accessibility and cellular localization require further study. Unlike long-acting GLP-1 receptor agonists, this approach aims to recruit endogenous enteroendocrine secretion. The magnitude and physiological importance of such a response remain uncertain. Existing reviews address extraoral taste receptors, gut chemosensing, and hormone release broadly [20,21,22,23]. However, studies linking natural bitter compounds (NBCs) to TAS2R signaling and GLP-1 secretion remain fragmented across ligands, receptor subtypes, experimental models, and secretory endpoints. A compound-specific assessment is therefore needed to establish the strength of the causal evidence.

To address this gap, we develop a compound- and model-resolved evidence map. The map distinguishes predicted ligand binding from functional receptor activation and evaluates whether receptor-directed perturbation changes extracellular GLP-1 secretion in the same model. It identifies the links that have been tested and the causal steps that remain unresolved. This distinction is important because changes in proglucagon expression, intracellular calcium signaling, or systemic metabolic outcomes cannot substitute for receptor-dependent GLP-1 secretion.

This narrative review was updated through 23 October 2026 using PubMed, Web of Science, Google Scholar, Scopus, and publisher-hosted full-text journal archives. Search concepts paired “bitter taste receptor” or “TAS2R” with “GLP-1”, “enteroendocrine”, “natural bitter compound”, or individual ligand names. Additional targeted searches covered intestinal organoids, microbiota, tuft-cell immunity, and vagal signaling. Original mechanistic reports were prioritized when the tested agent, receptor subtype, experimental species/model, receptor perturbation, and GLP-1 endpoint could be identified. Structural studies, human tissue studies, and clinical reports were retained to evaluate context and translation. General microbiota, immune, and neural studies were not counted as direct evidence of an NBC-TAS2R-GLP-1 pathway. This targeted, non-systematic approach was intended to appraise causal strength rather than estimate pooled effects.

“Receptor-supported” denotes a measured extracellular GLP-1 response reduced by subtype-directed genetic or receptor-focused pharmacological perturbation in the same ligand and model setting. This supports functional receptor involvement without establishing physical binding. “Indirect” denotes a demonstrated physiological association or shared signaling process without the matched receptor-dependent secretory test. “Hypothetical” denotes a proposed sequential link that has not been tested. Computational binding predictions and functional activation of individually expressed receptors are reported separately. Neither is treated as evidence of receptor-dependent GLP-1 secretion without a corresponding secretory experiment. Changes in proglucagon gene (GCG) expression or intracellular GLP-1 abundance are distinguished from hormone measured in culture supernatant or plasma. Active and total forms are specified when the source methods permit.

## 2. Molecular Basis and Expression Profile of Intestinal TAS2Rs

### 2.1. TAS2R Gene Family and Structural Characteristics

**Figure 2:**
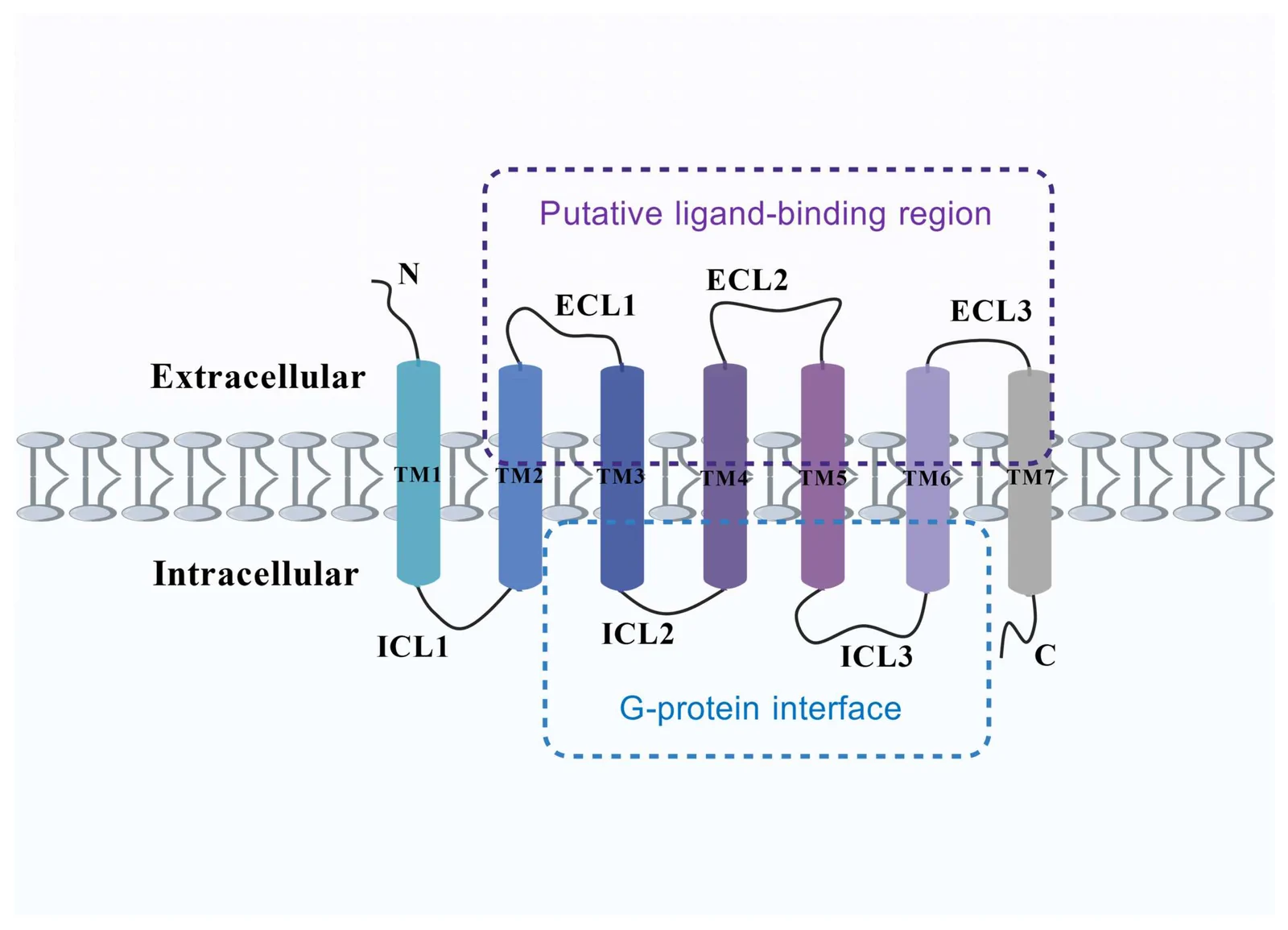
Schematic representation of structural features implicated in ligand recognition and G-protein coupling across structurally characterized human TAS2Rs. The depicted regions represent a synthesis of available structural studies and should not be interpreted as a universal binding architecture for all TAS2R subtypes. TM denotes a transmembrane helix, ECL an extracellular loop, and ICL an intracellular loop. Created with BioGDP.com [24].

TAS2Rs are seven-transmembrane GPCRs with distinctive sequence features (Figure 2) [25]. Their conserved transmembrane architecture contributes to ligand recognition and downstream signal transduction. Notably, TAS2R subtypes differ substantially in their ligand-receptive ranges [26,27,28]. Human TAS2Rs recognize a chemically diverse ligand repertoire [26], supporting their potential role as dietary chemical sensors.

Recent cryo-electron microscopy (cryo-EM) studies have revealed subtype-specific modes of ligand recognition. TAS2R46 can pre-couple to Gα-gustducin through an HPFIL-containing region spanning transmembrane helix 3 (TM3) and intracellular loop 2 (ICL2) [29]. TAS2R14 can engage ligands through distinct extracellular and intracellular regions [30,31,32,33,34], whereas TAS2R16 recognizes selected β-glucosides [35]. These observations derive from the structurally characterized subtypes and their studied ligands. They do not establish the binding pockets, coupling preferences, or functional activation of TAS2R38, TAS2R50, or other NBC-associated subtypes in intestinal L-cells. Docking against a predicted receptor model therefore remains a hypothesis about binding geometry rather than evidence of receptor activation.

Despite recent structural advances, high-resolution information remains unavailable for many TAS2R subtypes implicated in enteroendocrine signaling, limiting mechanistic interpretation of subtype-selective ligand recognition.

### 2.2. Cell-Type- and Region-Specific Expression of TAS2Rs in the Intestine

Intestinal TAS2Rs exhibit cell-type- and region-specific expression patterns, which may contribute to diverse chemosensory functions in the gut. Multiple TAS2Rs are expressed across intestinal epithelial cell populations [23]. Activation of some intestinal bitter taste receptors may alter the secretion of intestinal endocrine hormones in experimental models [36]. TAS2R-related chemosensory pathways have also been identified in tuft cells and have been associated with interleukin-25 (IL-25)-mediated type 2 immune responses [37,38,39]. Tas2r-associated signaling can participate in murine intestinal tuft-cell responses [38]. In addition, human primary jejunal crypts exhibit TAS2R-dependent innate immune responses [40].

Bitter-sensing machinery has been detected in intestinal epithelial populations, but regional receptor expression alone does not demonstrate a secretory response in native L-cells. Human ileal organoids contain functionally characterized GLP-1-secreting L-cells [41], whereas primary jejunal preparations from patients with obesity have shown bitter-stimulus-associated changes in growth differentiation factor 15 (GDF15) and GLP-1 expression through taste or motilin receptor-related mechanisms [42]. The latter study addresses expression and should not be cited as proof of increased secreted GLP-1 or of a complete TAS2R-dependent secretory mechanism. Moreover, intestinal segment, donor genotype, and disease state may change the apparent response [40,41,42,43].

Experimental models differ in what they can establish. NCI-H716 is a human colorectal carcinoma-derived cell line that can acquire an enteroendocrine phenotype. Caco-2 is primarily an absorptive intestinal epithelial carcinoma model rather than a native L-cell preparation, while HuTu-80 is a human duodenal carcinoma-derived model. All can provide tractable receptor-perturbation or hormone-release assays, but tumor origin, culture-dependent receptor expression, and incomplete cellular specialization limit extrapolation to human intestinal physiology [44,45,46,47,48,49]. Mouse STC-1 cells and rodent experiments add complementary evidence, but mouse Tas2r repertoires and endocrine physiology cannot be assumed to match human TAS2Rs [48,49,50].

Primary human intestinal tissue and donor-derived organoids can test whether cell-line findings persist in a more relevant epithelium [41,42,43,51,52,53]. However, organoids differ in L-cell abundance, apical accessibility, differentiation state, and donor sampling. Conventional epithelial organoids also lack intact vascular, neural, immune, and microbial compartments. Humanized animals may assist subtype testing but cannot replace direct human measurements. Existing berberine clinical trials evaluate metabolic outcomes, not matched intestinal TAS2R perturbation or target-site GLP-1 secretion [54,55,56,57]. Consequently, current human evidence supports the feasibility of studying this biology, not clinical proof of an NBC-TAS2R-GLP-1 mechanism.

### 2.3. Diversity and Subtype Specificity in Ligand Recognition

TAS2R ligand repertoires vary substantially. A single receptor may recognize many bitter molecules, while an individual ligand may activate several receptor subtypes [26,27,28]. Ligand recognition depends on receptor- and ligand-specific structural features [29,30,31,32,33,34,35]. TAS2R14 has a broad receptive range, whereas TAS2R16 recognizes selected β-glucosides through a more constrained pocket [26,35]. Some human TAS2R subtypes remain orphan receptors without experimentally validated agonists. Other subtypes have known agonists but have not been comprehensively characterized. These situations should not be described interchangeably.

The incomplete understanding of subtype-specific ligand recognition also limits rational development of TAS2R ligands with improved receptor selectivity and tissue-relevant activity. Integrating computational ligand prioritization with experimental validation in human-relevant models may facilitate identification of subtype-selective TAS2R ligands.

## 3. Interaction Mechanisms Between NBCs and TAS2Rs

### 3.1. Chemical Diversity of NBCs Relevant to TAS2R Signaling

**Figure 3:**
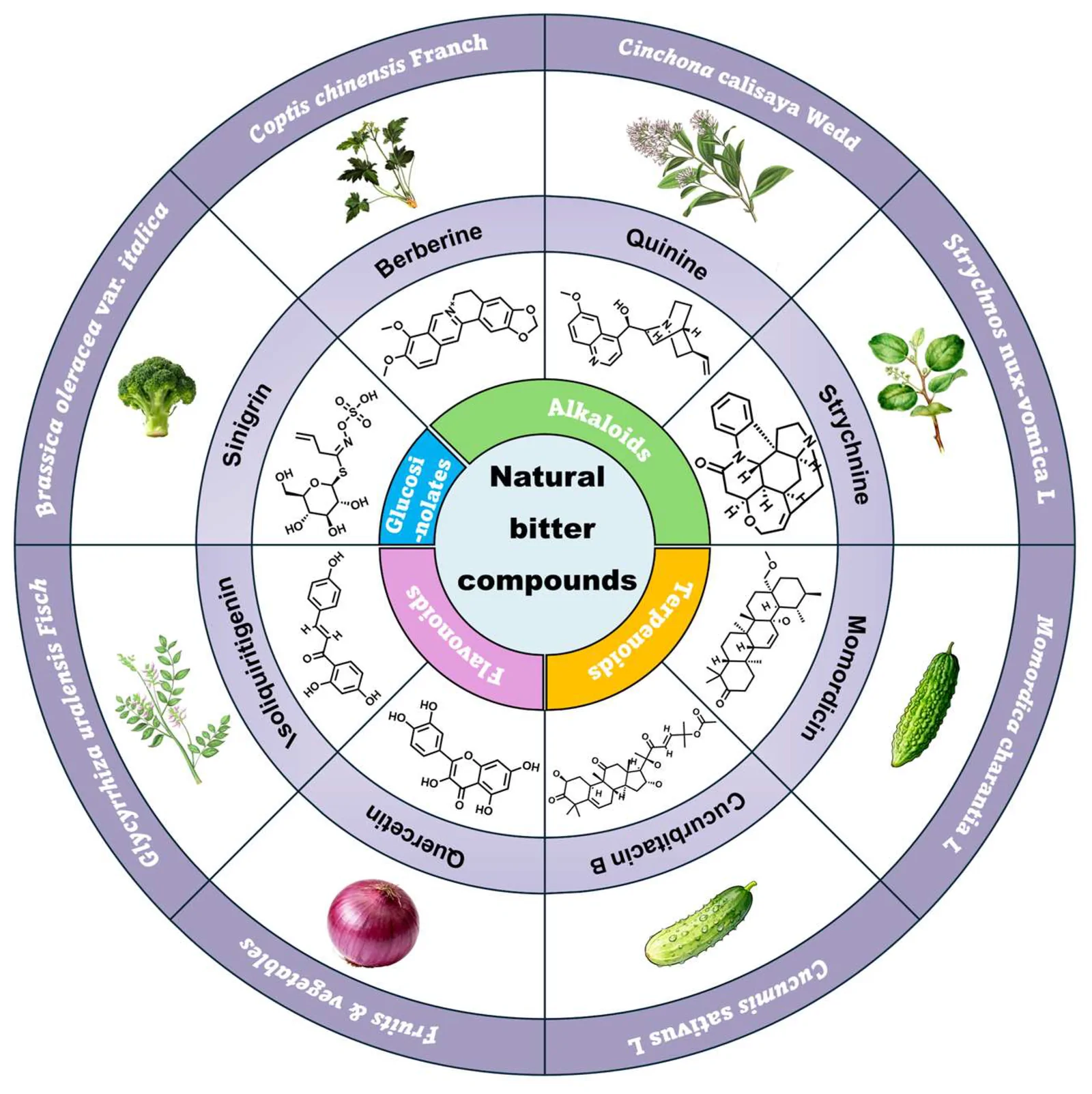
Chemical diversity and representative natural sources of NBCs investigated in relation to TAS2R signaling. The compounds shown represent major structural classes of bitter natural products rather than experimentally validated ligands for every depicted TAS2R subtype. Created with BioGDP.com [24].

For the purposes of this review, NBCs encompass chemically defined bitter constituents from foods or plants and bitter botanical preparations examined in relation to intestinal chemosensing (Figure 3). They include flavonoids, alkaloids, terpenoids, glycosides, and multicomponent extracts, but these materials differ markedly in purity, exposure, and receptor validation. A bitter taste, a computational receptor prediction, and a GLP-1 response are distinct observations. None alone assigns a specific TAS2R subtype to an agent.

We included natural-source agents when a primary study reported a TAS2R-related receptor assay, receptor perturbation, or enteroendocrine GLP-1 endpoint. The table distinguishes dietary-source constituents tested as purified molecules (e.g., quercetin and piperine), other purified phytochemicals examined largely at experimental/pharmacological concentrations (e.g., berberine, isosinensetin, and cucurbitacin B), a purified steviol glycoside with a bitter aftertaste (rebaudioside A), and botanical mixtures (hop extract). These are experimental-material categories, not claims of comparable dietary intake. Synthetic tastants such as denatonium are discussed as signaling comparators rather than counted as NBCs. Mixture effects cannot be assigned to an individual constituent without fractionation and receptor-specific testing.

### 3.2. Molecular Recognition of NBCs by TAS2Rs

NBC-TAS2R evidence ranges from computational docking to measured GLP-1 secretion after receptor-directed perturbation. Docking suggests a possible ligand-receptor pose but does not demonstrate activation. Functional activation in a heterologous receptor assay does not establish secretion by intestinal L-cells. A reduction in secretion after receptor knockdown supports receptor involvement in the tested model without proving direct physical binding. For example, rebaudioside A activates murine Tas2r108, Tas2r123, and Tas2r134 in transfected HEK293T assays at millimolar concentrations [48]. By contrast, piperine-TAS2R assignments from BitterX are computational predictions and must be distinguished from TAS2R14 knockdown results in Caco-2 cells [47].

For specific ligand-model combinations, matched receptor perturbation strengthens the inference of a TAS2R-dependent secretory response. TAS2R38-directed antibody or small interfering RNA (siRNA) attenuated berberine-evoked GLP-1 release [44], and TAS2R38 silencing reduced quercetin-evoked release in NCI-H716 cells [45]. Isosinensetin responses were reduced by TAS2R50 and G protein subunit alpha transducin 3 (GNAT3) silencing [46], while TAS2R14 knockdown reduced piperine-induced GLP-1 release in Caco-2 cells [47]. These results provide model-specific evidence of receptor involvement, not proof of subtype-selective binding in native human intestinal tissue.

Structural studies of other TAS2Rs illustrate why this distinction is important. TAS2R14 can accommodate ligands through unconventional recognition modes, including distinct extracellular and intracellular binding sites [31,32,33], whereas TAS2R16 exhibits more selective recognition of β-glucoside-containing ligands [35]. Such findings demonstrate substantial subtype-dependent diversity in TAS2R ligand recognition. However, high-resolution structures remain unavailable for many NBC–TAS2R pairs implicated in enteroendocrine signaling. Consequently, docking predictions or receptor-dependent functional responses should not be presented as structural confirmation of direct binding.

A rigorous interpretation of NBC–TAS2R interactions therefore requires separation of predicted binding, functional receptor activation, and receptor-dependent physiological responses. Combining structural biology, receptor mutagenesis, functional assays, and receptor-specific perturbation will help establish direct ligand–receptor relationships and define molecular determinants of subtype selectivity.

### 3.3. TAS2R-Associated Signaling in Enteroendocrine Cells

The canonical gustatory cascade provides a reference rather than a universal mechanism for all intestinal TAS2R subtypes. In taste cells, activation of TAS2R-associated G proteins releases G protein βγ subunits (Gβγ), which activate phospholipase C beta 2 (PLCβ2). The resulting inositol 1,4,5-trisphosphate (IP_3_) signal mobilizes intracellular Ca^2+^ and contributes to downstream excitability and secretion [18,58,59]. Related components are present in some enteroendocrine models [60,61]. Denatonium-induced GLP-1 release in NCI-H716 cells involves Gβγ, phospholipase C (PLC), inositol 1,4,5-trisphosphate receptor (IP_3_R), and Ca^2+^ [62]. Berberine responses involve TAS2R38 and PLC but are not blocked by inhibition of transient receptor potential melastatin 5 (TRPM5) [44,63]. Isosinensetin-induced release involves TAS2R50, GNAT3, PLC, and Ca^2+^ [46]. Piperine-induced secretion is reduced by TAS2R14 knockdown and inhibitors of Gβγ/PLC signaling in Caco-2 cells [47]. Cucurbitacin B has also been linked to Gβγ/PLC/IP_3_R signaling and a pathway involving calcium/calmodulin-dependent protein kinase kinase β (CaMKKβ) and AMP-activated protein kinase (AMPK) in differentiated NCI-H716 cells [49]. In STC-1 cells, rebaudioside A-stimulated active GLP-1 release was sensitive to adenylyl-cyclase inhibition, although stimulation with 2.5 mM rebaudioside A did not produce a detectable intracellular Ca^2+^ rise [48]. The isosinensetin and piperine studies also reported increased GCG messenger RNA (mRNA), which is distinct from GLP-1 measured in culture supernatants [46,47]. These findings support recurrent PLC/Ca^2+^ signaling in selected models but do not establish a universal TAS2R-GNAT3-PLCβ2-TRPM5 pathway.

Importantly, neither Ca^2+^ mobilization nor individual canonical taste-signaling components are sufficient to infer GLP-1 secretion. In GLUTag cells, quinine increased intracellular Ca^2+^ but produced limited GLP-1 exocytosis because of effects on the cortical actin network. Elevation of cyclic adenosine monophosphate (cAMP) restored secretion [64]. Similarly, TRPM5 inhibition did not suppress berberine-induced GLP-1 secretion in NCI-H716 cells [44], indicating that TRPM5 is not universally required. TAS2R signaling should therefore be viewed as a set of partially shared signaling modules rather than a single invariant TAS2R–gustducin–PLCβ2–TRPM5 pathway.

**Figure 4:**
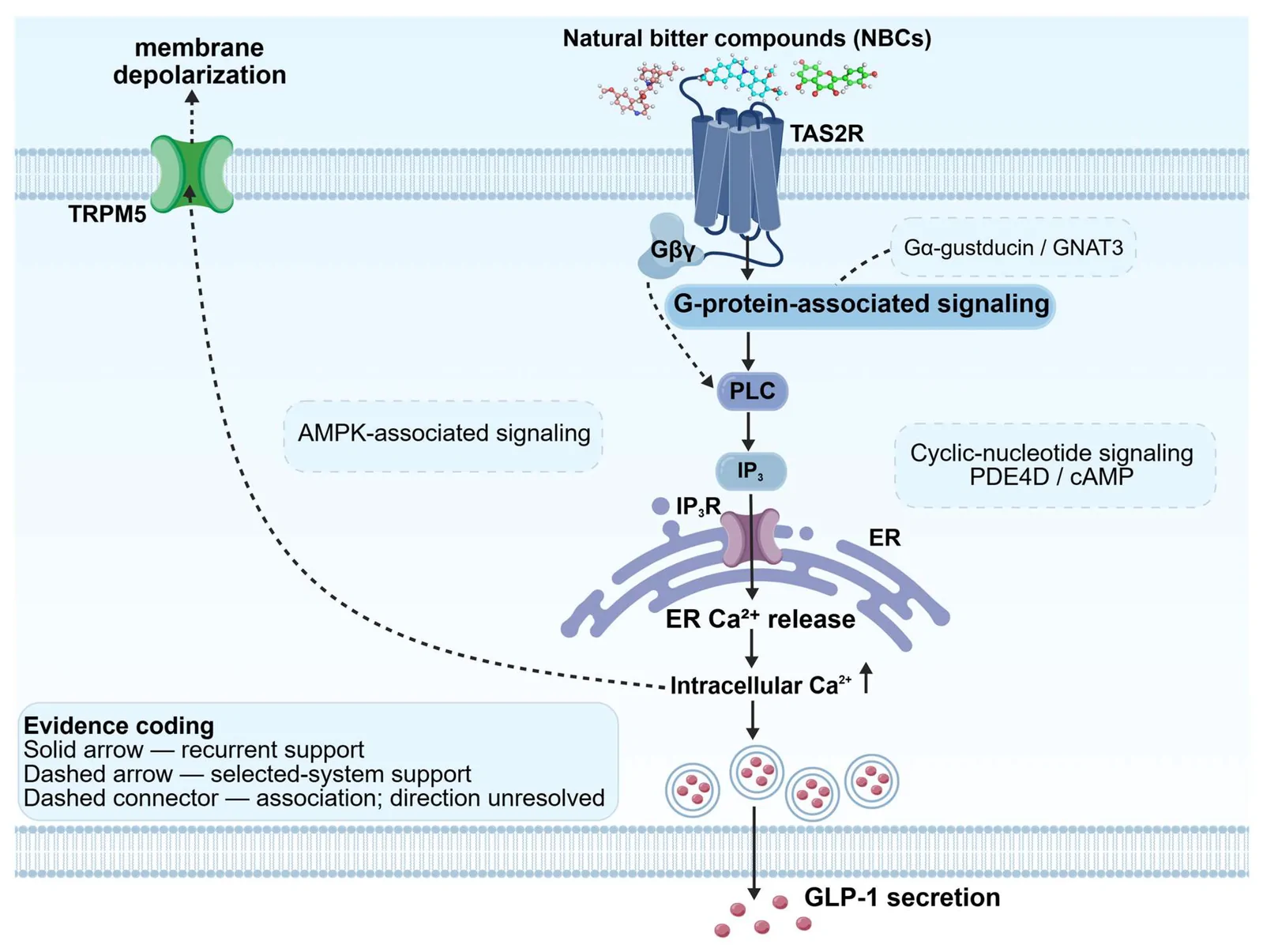
Evidence-weighted signaling modules implicated in TAS2R-associated GLP-1 secretion. Across the reviewed NBC–TAS2R experimental systems, PLC-dependent signaling and intracellular Ca^2+^ mobilization represent the most recurrently supported downstream modules. Gα-gustducin/GNAT3 and Gβγ contribute in selected experimental systems, whereas TRPM5-dependent depolarization, cyclic-nucleotide signaling, and AMPK-associated signaling show greater compound- or model dependence. The depicted modules integrate evidence from different compounds, receptor subtypes, and experimental systems and therefore should not be interpreted as a single invariant or sequentially validated signaling pathway. ER denotes endoplasmic reticulum, and PDE4D denotes phosphodiesterase 4D. Solid arrows indicate recurrently supported links, whereas dashed arrows indicate links supported in selected experimental systems. Dashed connectors without arrowheads indicate associations for which causal direction has not been established. The symbol ↑ denotes an increase. Colors distinguish schematic components and do not indicate evidence strength. Created with BioGDP.com [24].

Overall, PLC-associated signaling and IP_3_-dependent Ca^2+^ mobilization recur in several ligand-model combinations, whereas TRPM5, cAMP-associated signaling, protein kinase C (PKC), and AMPK show more context-specific involvement [44,46,47,48,49,62,64] (Figure 4). Receptor perturbation and assay specificity must be considered alongside extracellular GLP-1 measurements. An intracellular Ca^2+^ signal or higher GCG expression alone cannot establish hormone secretion. Whether the assay measured active or total GLP-1 is not consistently documented across studies.

## 4. An Evidence-Weighted Framework for TAS2R-Associated GLP-1 Regulation

The evidence-weighted framework distinguishes a receptor-supported enteroendocrine core from three possible modulatory interfaces. The core requires a reduction in measured NBC-evoked GLP-1 secretion after receptor-directed perturbation in the same model. Such a result supports functional dependence without proving direct ligand binding. Microbiota-associated, immune–endocrine, and gut–brain findings are classified as indirect when the matched NBC-TAS2R-GLP-1 test is absent. An untested sequential link is classified as hypothetical. The proposed interfaces therefore identify evidence gaps rather than established mechanisms equivalent to the core.

### 4.1. Core Mechanism: Enteroendocrine TAS2R-Associated Signaling

Several studies support a receptor-dependent secretory response in defined cellular models (Table 1). In human NCI-H716 cells, TAS2R38 perturbation attenuated berberine- and quercetin-associated GLP-1 release [44,45], and TAS2R50/GNAT3 silencing reduced isosinensetin-associated release [46]. In human Caco-2 cells cultured under high-glucose conditions, TAS2R14 short hairpin RNA (shRNA) attenuated piperine-evoked GLP-1(7–36) release, although this carcinoma model is not equivalent to a native human L-cell [47]. In murine STC-1 cells, Tas2r120/Tas2r138 knockdown reduced responses to a multicomponent hop extract [50]. The extract also reduced dipeptidyl peptidase-4 (DPP-4) activity (approximately 20% at 300 µg/mL), so its measured extracellular active GLP-1 signal may reflect both increased release and reduced peptide degradation [50].

Rebaudioside A illustrates why evidence levels must remain separate. In human HuTu-80 cells, gamma-aminobutyric acid (GABA) at 1 µM reduced rebaudioside A-stimulated total GLP-1 release by approximately 21%. The TRPM5 inhibitor triphenylphosphine oxide (TPPO) at 50 µM reduced release by approximately 27%, whereas 6-methoxyflavanone enhanced rather than blocked the response [48]. These findings suggest TAS2R4/TRPM5 involvement but do not provide subtype-specific genetic evidence. Rebaudioside A also activated murine Tas2r108, Tas2r123, and Tas2r134 in transfected HEK293T cells. The necessity of these receptors for GLP-1 secretion was not tested in STC-1 cells [48]. Cucurbitacin B provides a different evidence combination. TAS2R10 and GNAT3 siRNA attenuated extracellular GLP-1 release in differentiated NCI-H716 cells, while mouse experiments support Gα-gustducin dependence without assigning an individual murine Tas2r subtype in vivo [49].

The core evidence is therefore receptor-supported at the level of selected experimental secretory responses, but is not uniformly supported by direct structural binding data or by primary human L-cell validation. Assay endpoints also differ. Some studies quantify GLP-1(7–36) or report active or total GLP-1, whereas others do not verify the molecular form measured in extracellular samples (Table 1). These distinctions, together with unmeasured intestinal target-site concentrations, prevent simple ranking of candidate agents by in vitro response magnitude.

Other bitter agents, including quinine and extracts of Anemarrhena asphodeloides and Gentiana scabra, have been linked to GLP-1 exocytosis or release in experimental models [64,65,66]. Where the receptor subtype has not been causally tested, such findings support bitter-responsive enteroendocrine physiology but not a validated NBC-TAS2R pair. Changes in hormone precursor expression or intracellular signaling are described separately from extracellular secretion.

### 4.2. Microbiota-Associated Modulation

The microbiota-associated interface remains indirect. Microbial remodeling and metabolite-responsive enteroendocrine signaling have been documented in separate experimental contexts, but a shared intestinal TAS2R mechanism has not been established. Berberine alters microbial composition and bile-acid metabolism in experimental and clinical settings [54,67,68]. But these changes have not been shown to modify TAS2R-dependent GLP-1 release. Microbiota-derived short-chain fatty acids (SCFAs) contribute to intestinal physiology and host metabolic regulation [69]. These physiological effects do not establish a TAS2R-dependent mechanism of GLP-1 secretion. SCFAs can stimulate GLP-1 secretion through free fatty acid receptor 2 (FFAR2) [70]. Bile acids can also act through G protein-coupled bile acid receptor 1 (GPBAR1), also known as Takeda G protein-coupled receptor 5 (TGR5) [71,72]. These non-TAS2R pathways provide alternative explanations rather than evidence for an NBC-TAS2R-microbiota-L-cell chain. A key unresolved question is whether a defined microbial or NBC-derived metabolite changes a TAS2R-dependent GLP-1 response. This would require receptor-specific loss of function and rescue, direct measurement of extracellular GLP-1, and parallel FFAR2/GPBAR1 controls under matched exposure conditions.

### 4.3. Immune–Endocrine Interactions as a Potential Modulatory Interface

The immune–endocrine interface is a hypothesis requiring sequential testing. Tuft cells express chemosensory signaling components, including GNAT3, PLCβ2, IP_3_R2, and TRPM5. These cells can also engage IL-25-associated type 2 immune responses [37,38,39,73]. Murine tuft-cell Tas2r signaling and human intestinal epithelial immune responses have also been reported [38,40]. However, these components can also be engaged by non-TAS2R chemosensors, so shared signaling machinery cannot by itself identify a specific NBC-activated TAS2R [73]. Moreover, these observations do not demonstrate that an immune mediator subsequently drives TAS2R-dependent GLP-1 secretion by L-cells. Cell-type-specific receptor perturbation, mediator blockade, and a secreted GLP-1 endpoint are needed to establish this sequence. In epithelial–immune co-cultures, blockade and rescue of the candidate mediator would help distinguish a necessary signaling intermediate from a correlated immune phenotype.

### 4.4. Gut–Brain Communication as a Downstream and Potential Modulatory Interface

The gut–brain interface must distinguish downstream transmission from upstream control of secretion. GLP-1-associated vagal and brainstem signaling is established in general [74,75], and enterochromaffin–neural communication and tuft-cell proximity to nerves support intestinal sensory connectivity [76,77]. These findings do not show that a specified NBC acts through an intestinal TAS2R to recruit a neural feedback circuit that controls L-cell GLP-1 release. Neural signaling is therefore best regarded as a supported downstream consequence of GLP-1 release and a possible, but unproven, upstream modifier of NBC-associated secretion. To establish an upstream role, selective neural manipulation should be combined with subtype-specific TAS2R perturbation and time-resolved measurement of extracellular GLP-1, distinguishing altered hormone release from downstream signal transmission.

### 4.5. Evidence Status, Missing Links, and Testable Predictions

Table 2 provides a comparative evidence map. Receptor-directed loss of function supports the enteroendocrine core in selected transformed cell systems [44,45,46,47,49,50], but native human receptor selectivity, secreted hormone forms, and local exposure remain incompletely resolved. By contrast, microbiota-dependent GLP-1 regulation through FFAR2/GPBAR1 [70,71,72], tuft-cell immune signaling [37,38,39,40,73], and vagal responses to GLP-1 [74,75] are established in their own contexts. None independently demonstrates the NBC-TAS2R-dependent causal chain proposed for an interface.

The microbiota hypothesis requires evidence that a defined metabolite changes TAS2R-dependent GLP-1 release. The proposed immune link requires a mediator that connects TAS2R activation to L-cell secretion. A neural feedback mechanism would need to change NBC-induced secretion rather than merely transmit its downstream signal. Tests should combine defined exposures with subtype-specific receptor perturbation, extracellular GLP-1 measurements, and pathway controls (Table 2).

This framework advances previous reviews by mapping what has been measured, which receptor-dependent links have been tested, and which attractive biological connections remain hypotheses. It does not assign equivalent mechanistic certainty to all four components.

## 5. Translational Barriers to TAS2R-Targeted GLP-1 Modulation

Translational evaluation of NBC-induced intestinal TAS2R signaling requires more than an in vitro GLP-1 response. The major unresolved variables are local luminal/mucosal exposure, receptor-subtype selectivity, the form and magnitude of secreted GLP-1, and donor-to-donor response variability. These are prerequisites for evaluating nutritional or pharmacological relevance, not evidence that clinical efficacy has already been achieved.

### 5.1. Target-Site Exposure and Pharmacokinetic Uncertainty

A critical question is whether oral NBC exposure produces sufficient free compound at the relevant intestinal epithelium to engage a receptor. For example, berberine has low systemic oral bioavailability [78,79], but plasma levels alone do not establish its luminal or mucosal concentrations. A high nominal luminal concentration also does not demonstrate that free compound reaches an accessible epithelial receptor. The nominal in vitro concentrations ranged from 1 to 200 µM for berberine [44] and from 25 to 400 µM for quercetin [45]. Other studies tested 40 or 80 µM isosinensetin [46], 2 to 50 µM piperine [47], and 100 or 300 µg/mL hop extract [50]. Rebaudioside A was tested at 0.5 to 1 mM in mouse STC-1 secretion assays [48] (Table 1). The highest quercetin concentration was associated with reduced cell viability [45]. Assay durations and GLP-1 forms also differed. Rebaudioside A, for example, was assessed as active GLP-1 in STC-1 cells but as total GLP-1 in HuTu-80 cells [48]. These experimental concentrations should not be equated with exposure at human intestinal TAS2Rs after ordinary dietary intake.

Future work should quantify parent compounds, unbound fractions, and relevant metabolites in the lumen and at the mucosal surface, alongside receptor dose–response curves and extracellular active/total GLP-1 measurements. Absorption, transit, solubility, metabolism, and epithelial polarity can make nominal culture-medium concentration a poor proxy for receptor exposure in vivo. When receptor localization or epithelial access is uncertain, apical and basolateral stimulation should be compared separately. A physiologically credible mechanism requires overlap between measured target-site exposure and a receptor-dependent secretory concentration range, not merely low or high plasma bioavailability.

### 5.2. Receptor Selectivity, Oral Bitterness, and Off-Target Activation

Most bitter agents are not selective for a single TAS2R subtype, and structural results from TAS2R14, TAS2R16, or TAS2R46 cannot be generalized to all intestinal receptors [26,27,28,29,30,31,32,33,34,35]. Subtype assignment should therefore combine functional receptor-panel counter-screening with orthogonal antagonism or genetic loss of function, rather than rely on one docking score or a broadly acting inhibitor. Possible engagement of other chemosensors and non-TAS2R targets must be evaluated when interpreting GLP-1 secretion. The incomplete and sometimes directionally unexpected pharmacological modulation of rebaudioside A responses illustrates why antagonist results require genetic corroboration [48].

Receptor selectivity also has practical implications. Activation of oral TAS2Rs can produce undesirable bitterness and may limit the acceptability of bitter-compound interventions. Moreover, TAS2Rs are expressed in multiple extraoral tissues, raising the possibility that broadly active compounds could engage receptors outside the intended intestinal target [21,40]. The physiological relevance of such extra-intestinal activation depends on compound exposure, receptor subtype, and tissue context and remains insufficiently characterized for most NBCs. Accordingly, GLP-1 secretion alone is not sufficient to establish suitability for TAS2R-targeted intervention. Receptor-subtype dependence and physiologically relevant exposure should also be considered.

### 5.3. Interindividual Variability and Response Heterogeneity

TAS2R sequence variation and tissue context may contribute to response heterogeneity. TAS2R38 is a well-studied example of genotype-dependent bitter perception [80,81], and human genetic associations link some TAS2R38 variants with postprandial glycemia [82]. Neither association demonstrates genotype-dependent GLP-1 secretion after a defined NBC. Human jejunal studies also suggest variation in bitter-stimulus-related GDF15/GLP-1 expression across receptor and genetic contexts, but expression changes are not interchangeable with secretion [42].

Variability may also arise from differences in intestinal physiology, disease state, diet, and microbiome-dependent metabolism of NBCs. These factors can alter compound exposure and metabolite profiles and may therefore contribute to heterogeneous responses. At present, however, no combination of TAS2R genotype, microbiome composition, or metabolic phenotype has been prospectively validated as a predictor of NBC-induced, TAS2R-dependent GLP-1 secretion. Interindividual variability should therefore be treated primarily as a source of mechanistic uncertainty rather than as an established basis for precision nutrition. Donor-stratified intestinal studies should record these variables before proposing genotype- or microbiome-based responder groups.

## 6. Priorities for Mechanistic and Human Validation

**Figure 5:**
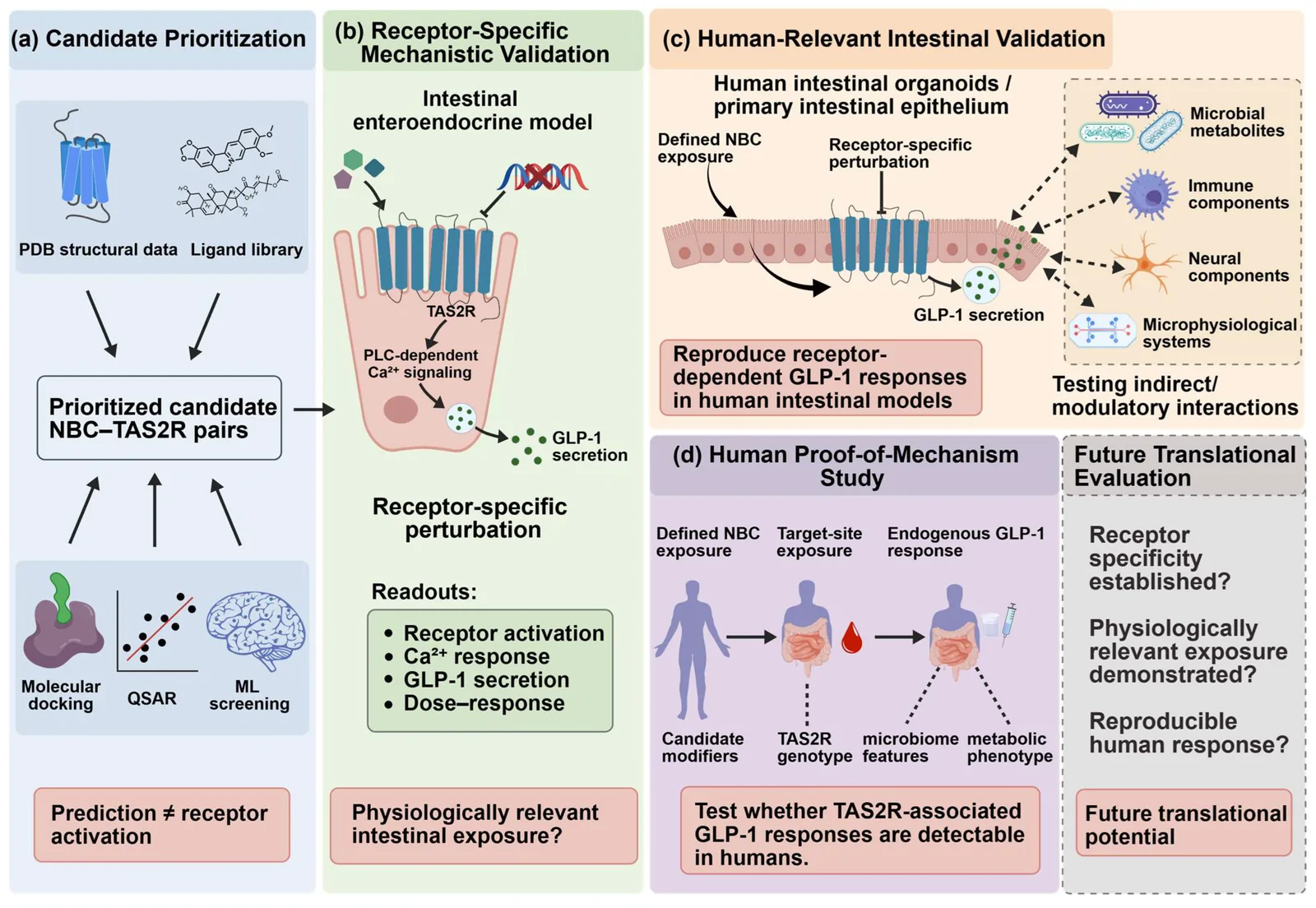
Framework for mechanistic and human validation of TAS2R-associated GLP-1 regulation. Panel (**a**) shows how computational methods can prioritize candidate NBC–TAS2R pairs. Panel (**b**) illustrates receptor-specific testing of GLP-1 secretion at plausible intestinal exposures. Panel (**c**) depicts validation in human-derived intestinal epithelium and investigation of proposed modulatory interfaces. Panel (**d**) outlines prospective human studies of endogenous GLP-1 responses. The framework describes increasing evidence strength rather than a mandatory development sequence. PDB denotes the Protein Data Bank. QSAR refers to quantitative structure–activity relationship modeling, and ML denotes machine learning. Created with BioGDP.com [24].

Future work should first determine whether candidate compounds reach relevant intestinal sites at concentrations that activate a defined TAS2R. Receptor-dependent GLP-1 secretion must then be reproduced in human-derived intestinal epithelium before the mechanism is tested prospectively in humans. Table 2 identifies the corresponding evidence gaps, and Figure 5 illustrates the proposed validation sequence.

### 6.1. Establishing Physiologically Relevant Intestinal Exposure

Mechanistic experiments should relate receptor activation and extracellular GLP-1 secretion to measured free NBC concentrations at relevant intestinal sites, rather than infer luminal activity from systemic pharmacokinetics. For poorly absorbed agents such as berberine, this requires simultaneous measurement of parent compound and metabolites, epithelial access, and receptor-specific dose–response under realistic exposure conditions [78,79]. Where direct sampling is infeasible, modeled target-site concentrations should be explicitly labeled as estimates and examined across plausible exposure assumptions. Dose–response studies should measure cell viability alongside active GLP-1 secretion, especially at upper concentrations such as 400 µM quercetin, where reduced viability was reported [45]. When only active GLP-1 is measured, parallel DPP-4 activity or peptide-recovery controls are also needed to distinguish enhanced secretion from reduced degradation, particularly for botanical mixtures [50].

Formulation studies may change intestinal delivery [83], but improved plasma bioavailability or glycemic outcomes alone cannot demonstrate enhanced TAS2R engagement. Any formulation claim should be supported by a change in intestinal target-site exposure accompanied by a reproducible, receptor-dependent GLP-1 response.

### 6.2. Structure-Guided and Computational Prioritization of TAS2R Ligands

Structures of selected TAS2R subtypes now permit hypothesis-driven ligand prioritization [29,30,31,32,33,34,35]. Docking, quantitative structure–activity relationship (QSAR) models, and machine-learning screens can nominate candidates [84,85,86], but their scores do not constitute evidence of receptor activation or physiological efficacy.

A candidate predicted in silico should first show a concentration-dependent functional response in cells expressing the individual human TAS2R, with receptor-negative controls and counter-screening against other TAS2Rs. Orthogonal receptor mutagenesis or binding experiments can test the proposed recognition mode. Next, receptor knockdown or knockout followed by rescue should demonstrate dependence of measured extracellular GLP-1 release in a relevant enteroendocrine model. Proglucagon mRNA and Ca^2+^ signals should be reported as separate endpoints. The assay must specify whether active GLP-1, total GLP-1, or GLP-1(7–36) was measured and include exposure and viability controls. Finally, the response should be replicated in donor-derived human intestinal epithelium at measured target-site concentrations. This sequence converts computational prioritization into testable mechanistic evidence without treating the prediction itself as proof.

### 6.3. Human-Relevant Models for Testing Unresolved Causal Links

Donor-derived human intestinal organoids and primary tissues can assess whether candidate receptor-dependent GLP-1 responses reproduce beyond transformed cell lines [41,51,52,53]. Ileal organoids containing functional L-cells are available [41], and single-cell analyses can help localize receptor expression and secretory phenotypes [53]. However, variable L-cell abundance, donor sampling, apical access, and missing stromal, immune, microbial, and neural compartments limit inference about intact physiology. Segment-matched primary tissues and multiple donors should therefore complement organoid studies.

For the core mechanism, matched TAS2R loss of function and rescue should be combined with active/total GLP-1 secretion assays and GCG-expression measurements. For an indirect interface, defined microbial metabolites, immune mediators, or neural elements can be added one at a time with alternative-receptor controls. Reproduction of a response in co-culture would establish model-level interaction, but not by itself prove its quantitative contribution in vivo.

Microphysiological systems may extend this approach by incorporating selected features of intestinal physiology, including fluid flow, mechanical forces, and interactions between epithelial and non-epithelial compartments [87,88,89]. Gut–liver platforms can additionally model aspects of intestinal–hepatic drug metabolism and may be useful for examining how NBC biotransformation alters intestinal exposure [90,91]. These systems are therefore most appropriately viewed as experimental platforms for testing defined mechanistic questions rather than as substitutes for human physiological validation.

### 6.4. Requirements for Future Human Validation

Human evidence is required to establish physiological importance. Randomized trials of berberine-containing interventions have reported metabolic, microbiome, or postprandial outcomes [55,56,57], but did not test a specified intestinal TAS2R-dependent GLP-1 mechanism. Clinical glycemic improvement therefore should not be treated as evidence of TAS2R activation or receptor-dependent GLP-1 secretion.

Prospective mechanism-oriented studies could use a prespecified, randomized crossover design comparing a chemically defined NBC with an appropriate control during a standardized meal challenge. The protocol should measure active and total GLP-1 over time, using appropriate DPP-4 stabilization during sample collection. Relevant metabolites, target-site or luminal exposure where feasible, and tolerability should also be assessed. TAS2R genotype and microbiome features may be evaluated as exploratory effect modifiers, but any association would require independent replication and should not be interpreted as proof of receptor causality without complementary mechanistic evidence.

Precision-nutrition or therapeutic claims remain premature until receptor specificity, plausible intestinal exposure, and reproducible human responses are demonstrated. The priority is mechanistic confirmation of endogenous GLP-1 modulation, not immediate recommendation of individual NBCs or doses. Together, these experiments provide a staged path from candidate ligand prediction to human mechanistic evidence (Figure 5). The stages represent evidentiary requirements rather than a guaranteed clinical-development sequence.

## 7. Conclusions and Future Perspectives

This review provides a compound- and model-specific assessment of how natural bitter agents may influence GLP-1 secretion through intestinal TAS2Rs. Receptor-directed perturbation supports secretory responses to several compounds in transformed cell lines, although direct binding and native human L-cell validation remain limited. PLC-associated Ca^2+^ signaling is common in some models but not universal. The cAMP-sensitive rebaudioside A response in STC-1 cells, for example, lacks matched subtype-specific loss-of-function evidence.

The proposed microbiota, immune, and neural interfaces remain plausible research directions rather than demonstrated NBC–TAS2R–GLP-1 pathways. Their principal unresolved causal steps are identified in Table 2.

Future work should prioritize receptor-specific loss of function and rescue at measured intestinal exposure levels. These experiments need to distinguish secreted active or total GLP-1 from precursor expression and should be reproduced in human-derived intestinal models. Prospective human studies will then be needed to determine whether this mechanism has physiological or therapeutic relevance.

## Figures and Tables

**Table 1:** Model- and species-resolved evidence for natural bitter agents, TAS2Rs, and GLP-1 endpoints, including tested exposures.

Agent/Category	Model/Species	Receptor Evidence	GLP-1 Endpoint	Tested Exposure	Ref.
Quercetin Purified dietary-source flavonoid	NCI-H716 Human	TAS2R38 siRNA reduced secreted GLP-1; docking was predictive, not a functional receptor assay.	GLP-1(7–36) ELISA; medium and intracellular levels. GCG/PC3 mRNA separately.	25–400 µM (2 h); reduced cell viability at 400 µM.	[45]
Isosinensetin Purified phytochemical	NCI-H716 Human	TAS2R50 and GNAT3 siRNA reduced secretion; PLC/IP_3_R perturbation.	Medium GLP-1(7–36) ELISA; GCG mRNA separately.	40, 80 µM (GLP-1, 1 h); 80 µM (mechanistic tests).	[46]
Berberine Purified alkaloid	NCI-H716 Human	TAS2R38 siRNA and receptor-directed antibody reduced release; PLC inhibition.	Active GLP-1 ELISA in medium.	1–200 µM (2 h); inhibitor assays: 100 µM.	[44]
Piperine Purified dietary-source alkaloid	Caco-2 Human	TAS2R14 shRNA reduced release; BitterX predictions are not direct activation.	Medium GLP-1(7–36) ELISA; GCG mRNA separately.	2, 10, 50 µM (GLP-1, 1 h; GCG/protein, 24 h).	[47]
Hop extract Botanical mixture	STC-1 Mouse	Tas2r120/Tas2r138 siRNA Reduced release; individual active constituent not assigned.	Active GLP-1 in medium; DPP-4 inhibition may also increase measured active GLP-1 concentrations. Ca^2+^ responses.	100, 300 µg/mL (2 h).	[50]
Rebaudioside A Purified steviol glycoside	HuTu-80 Human	GABA/TPPO partly inhibited release; 6-MF enhanced it; no TAS2R4 knockdown was performed.	Total GLP-1 in medium.	≥250 µM (2 h); 750 µM (inhibitor assays).	[48]
Rebaudioside A	STC-1 Mouse	cAMP-sensitive GLP-1 response; no matched subtype-specific knockdown in this cell model.	Active GLP-1 in medium.	0.001–1 mM; significant at 0.5–1 mM (2 h).	[48]
Rebaudioside A	HEK293T assay Mouse receptors	Direct functional activation of Tas2r108, Tas2r123, and Tas2r134.	Receptor Ca^2+^ assay; GLP-1 not measured.	1 and 3 mM (screen); up to 3 mM (dose–response).	[48]
Cucurbitacin B Purified terpenoid	NCI-H716 Human	TAS2R10 and GNAT3 siRNA attenuated release; TAS2R14 not verified by methods or plotted siRNA labels.	Medium GLP-1; form not specified; AMPK/PLC-linked responses.	10–100 µM (GLP-1, 30 min).	[49]
Cucurbitacin B	C57BL/6, db/db; Gnat3-null Mouse	Gα-gustducin-dependent in vivo response; individual murine Tas2r subtype not assigned.	Plasma GLP-1 by multiplex assay; form not specified.	0.1 mg/kg oral.	[49]

Human receptors are denoted TAS2R and mouse receptors Tas2r throughout the table. Receptor-dependent secretion means attenuation of measured extracellular GLP-1 after receptor-directed perturbation in the same model. Heterologous activation and computational prediction are separate endpoints. GLP-1(7–36) denotes the specified assay analyte, whereas “form not verified” means active/total specificity could not be established from the available methodological description. Tested concentrations are experimental bath concentrations or administered doses, not measured intestinal target-site exposures. ELISA denotes enzyme-linked immunosorbent assay, PC3 denotes prohormone convertase 3, and 6-MF denotes 6-methoxyflavanone.

**Table 2:** Comparative evidence map of established background mechanisms, missing NBC-TAS2R-GLP-1 links, and discriminating experiments.

Component/Evidence Level	Established Observation	Missing Causal Link	Discriminating Experiment
Core (receptor- supported) [44,45,46,47,49,50] Partial pharmacological support [48]	Subtype-directed perturbation reduces extracellular GLP-1 in transformed-cell models; rebaudioside A has only partial pharmacological support.	Direct binding, native human L-cell response, and realistic target-site exposure remain unresolved.	Test receptor loss of function and rescue in donor-derived ileal epithelium, measuring active and total GLP-1 at relevant exposure.
Microbiota interface Indirect evidence Hypothesis [54,67,68,69,70,71,72]	Berberine alters microbial and bile-acid profiles. FFAR2 and GPBAR1/TGR5 independently regulate GLP-1.	No matched microbial metabolite-TAS2R-dependent GLP-1 causal test.	Test a defined metabolite with TAS2R knockout and rescue, alongside FFAR2/GPBAR1 controls.
Immune–endocrine interface Indirect evidence Hypothesis [37,38,39,40,73]	Tuft-cell taste machinery and IL-25 immunity are supported in distinct models.	No sequential NBC-TAS2R-immune mediator-L-cell secretion chain.	Cell-specific TAS2R perturbation, mediator neutralization, and GLP-1 secretion in co-culture.
Gut–brain interface Downstream established Feedback hypothetical [74,75,76,77]	GLP-1 engages vagal circuits. Epithelial-neural contacts have also been described.	Neural transmission is not evidence that nerves regulate NBC-TAS2R-evoked secretion.	Pair intestinal TAS2R Perturbation with neural manipulation and timed GLP-1 readouts.

Evidence status refers specifically to a causal NBC-TAS2R-dependent GLP-1 response, not to the validity of general microbial, immune, or neural physiology. References identify the studies supporting each component. The listed experiments are proposed tests rather than completed validations.

## Data Availability

No new data were created or analyzed in this study. Data sharing is not applicable to this article.

## Abbreviations

The following abbreviations are used in this manuscript:

6-MF	6-methoxyflavanone
AMPK	AMP-activated protein kinase
cAMP	Cyclic adenosine monophosphate
ANP	Atrial natriuretic peptide
CaMKKβ	Calcium/calmodulin-dependent protein kinase kinase β
cryo-EM	Cryo-electron microscopy
DPP-4	Dipeptidyl peptidase-4
ECL	Extracellular loop
ELISA	Enzyme-linked immunosorbent assay
ER	Endoplasmic reticulum
FFAR2	Free fatty acid receptor 2
GABA	Gamma-aminobutyric acid
GCG	Proglucagon gene
GDF15	Growth differentiation factor 15
GI	Gastrointestinal
GLP-1	Glucagon-like peptide-1
GNAT3	G protein subunit alpha transducin 3
GPBAR1	G protein-coupled bile acid receptor 1
GPCRs	G protein-coupled receptors
Gβγ	G protein βγ subunits
ICL	Intracellular loop
IL-25	Interleukin-25
IP_3_	Inositol 1,4,5-trisphosphate
IP_3_R	Inositol 1,4,5-trisphosphate receptor
ML	Machine learning
mRNA	Messenger RNA
NBCs	Natural bitter compounds
PC3	Prohormone convertase 3
PDB	Protein Data Bank
PDE4D	Phosphodiesterase 4D
PKC	Protein kinase C
PLC	Phospholipase C
PLCβ2	Phospholipase C beta 2
QSAR	Quantitative structure–activity relationship
SCFAs	Short-chain fatty acids
shRNA	Short hairpin RNA
siRNA	Small interfering RNA
T2DM	Type 2 diabetes mellitus
TAS2R	Taste receptor type 2 (bitter taste receptor)
TGR5	Takeda G protein-coupled receptor 5
TM	Transmembrane helix
TPPO	Triphenylphosphine oxide
TRPM5	Transient receptor potential melastatin 5
